# A Review of Crystalline Multibridged Cyclophane Cages: Synthesis, Their Conformational Behavior, and Properties

**DOI:** 10.3390/molecules27207083

**Published:** 2022-10-20

**Authors:** Xing-Xing Zhang, Jian Li, Yun-Yin Niu

**Affiliations:** 1 Green Catalysis Center, College of Chemistry, Zhengzhou University, No. 100 Science Avenue, Zhengzhou 450001, China; 2 College of Ecology and Environment, Zhengzhou University, Zhengzhou 450001, China

**Keywords:** cyclophane, conformation, supramolecule, 3D molecule, NHC carbene, review

## Abstract

This paper reviews the most stable conformation of crystalline three-dimensional cyclophane (CP) achieved by self-assembling based on changing the type of aromatic compound or regulating the type and number of bridging groups. [3_n_]cyclophanes (CPs) were reported to form supramolecular compounds with bind organic, inorganic anions, or neutral molecules selectively. [3_n_]cyclophanes ([3_n_]CPs) have stronger donor capability relative to compound [2_n_]cyclophanes ([2_n_]CPs), and it is expected to be a new type of electron donor for the progress of fresh electron conductive materials. The synthesis, conformational behavior, and properties of crystalline multi-bridge rings are summarized and discussed.

## 1. Introduction

Supramolecular chemistry was first proposed by the “Father of Supramolecular Chemistry” Lehn based on Pedersen’s study of crown ethers [1,2,3,4], it is mainly the science of studying the system with specific functions formed by chemical substances through the intermolecular force of noncovalent bonds [5,6]. As an interdisciplinary subject, supramolecular science has made remarkable achievements in recent years [7,8]. It has great application potential in the fields of life science, environmental science, materials science, energy science, and medical science [9,10]. The designs and synthesis of artificial organic macrocyclic compounds with molecular recognition ability are one of the research hotspots in supramolecular chemistry [11,12]. As artificial receptors, cyclophanes are macrocyclic compounds formed by bridging multiple aromatic compounds [13,14]. By changing the type of aromatic compounds or regulating the type and quantity of bridging groups [15], cyclophanes are endowed with new self-assembly strategies and physical-chemical synergistic driving forces such as hydrogen bond, electrostatic interaction, hydrophobic interaction, π-π bond, metal ion coordination and so on [16,17,18]. Therefore, cyclophane and its derivatives, including crown ether, cyclodextrin, and calixarene, are becoming a very important class of host compounds in supramolecular chemistry [19,20,21,22,23,24]. Because cyclophane has multiple active sites and a unique cavity structure, it can selectively recognize guest molecules or ions [25,26,27,28,29,30]. It also has many properties such as optical properties, catalytic activity and drug activity [31,32], which gives it broad application prospects in the fields of chemical battery [33,34], electrochemical sensor [35], biomedical, [31] material science [36] and so on.

**Figure 1:**
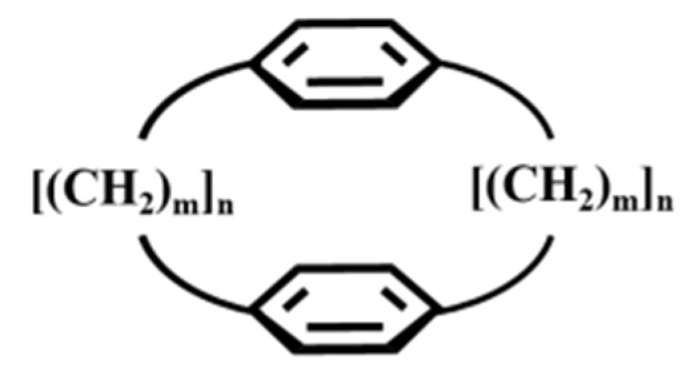
The structure diagram of [m_n_]cyclophane (m = n = 3–6).

**Figure 2:**
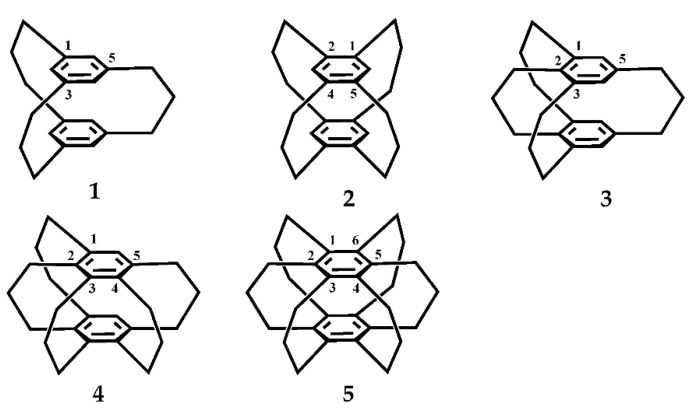
The structure diagrams of [3_3_](1,3,5)CP **1**, [3_4_](1,2,4,5)CP **2**, [3_4_](1,2,3,5)CP **3**, [3_5_](1,2,3,4,5)CP **4**, and [3_6_](1,2,3,4,5,6)CP **5**.

The term multiple-bridged cyclophane is understood to denote those with more than two bridges, as commonly used by [m_n_]cyclophane(m = n = 3–6) (Figure 1) to indicate poly bridge cyclopean, for example, [2_n_]CPs contain two benzene rings and three to six ethanol bridges and [3_n_]CPs contain two benzene rings and three to six propane bridges. Figure 2 shows the structural diagrams of [3_3_](1,3,5)CP, [3_4_](1,2,4,5)CP, [3_4_](1,2,3,5)CP, [3_5_](1,2,3,4,5)CP, [3_6_](1,2,3,4,5,6)CP [37], which look like some pinwheels with different blades [38,39]. In recent decades, with the synthesis of a large number of artificial cyclophanes [40,41,42,43], two-dimensional (2D) and three-dimensional (3D) multiple-bridged cyclophanes have a wide development trend and are widely used in the field of artificial macrocyclic compounds [44]. In the artificial bionic simulation experiment, it largely depends on whether an efficient active cage can be constructed and whether the main molecules with specific active cavities can be designed and synthesized [45,46,47]. Compared with two-dimensional planar cyclophane, three-dimensional cyclophane can build catalytic cages and form more valuable simulation systems, which is of great significance not only for the selective recognition of anions, amphoteric molecules, and other substances [48,49,50], but also for the catalysis of supramolecule [51]. Therefore, this review is based on the summary and progress of three-dimensional cyclophanes [52]. The focus is especially on the three-dimensional cyclophane cages in the crystalline state because the crystalline material has special properties such as anisotropy, definite melting point, and X-ray diffraction compared with the amorphous material [53,54].

## 2. Results and Discussion

### 2.1. Crystalline Three-Fold Bridged Cyclophanes

#### 2.1.1. Carbon Chain Three-Bridge Cage

**Figure 3:**
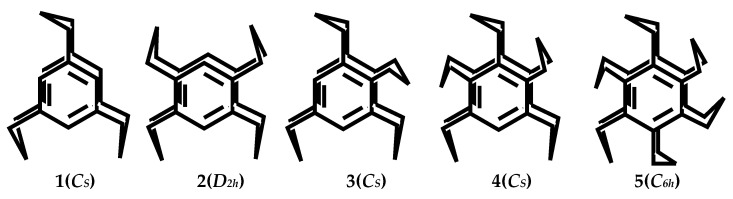
The most secure conformers of multibridged [3_n_]CPs (n = 3–6).

Carbon chain three-bridge cyclophanes [3_n_]CPs are flowing in solution, and among them, the flip process of Sanya methyl bridges causes their conformational isomerism. The most secure conformers are summed up in Figure 3 [37].

**Figure 4:**
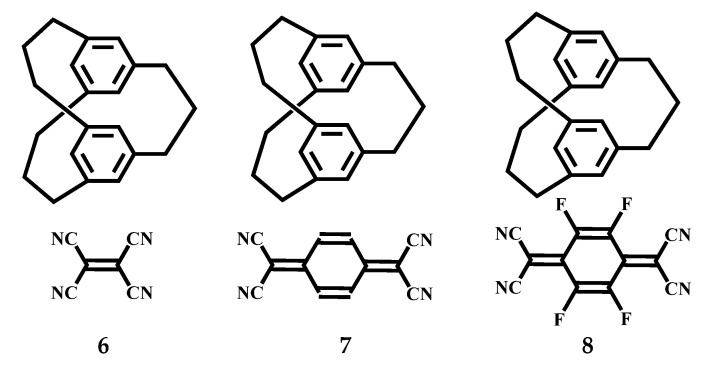
The structures of [3_3_]CPs -TCNE, -TCNQ, and -TCNQ-F4 complexes **6**, **7**, and **8**.

**Figure 5:**
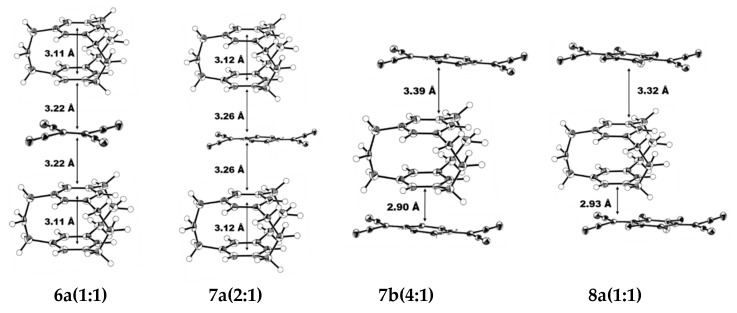
ORTEP drawings of complexes **6**, **7**, and **8** at −180 °C (the probability of thermal ellipsoid is 50%).

By making use of the strong ability of [3_n_]CPs to give electrons, Mikio et al. synthesized the CT (charge-transfer) compounds with TCNE (tetracyanoethylene), TCNQ (Tetracyanoquinodimethane), and TCNQ-F4. [3_3_](1,3,5) CP-TCNE (1:1) compound **6** (Figure 4) was synthesized at −180 °C [37]. In its crystal structure, each TCNE was arranged as a sandwich between two cyclophane molecules, located on or near the center of symmetry, which resulted in a compound (1:1) with alternating D-A stacking. The benzene ring in the compound is almost parallel to the TCNE molecule and the average distance between plane-to-plane is 3.22 Å. The average transannular distance between the two benzene rings is 3.11 Å (Figure 5). [3_3_](1,3,5)CP-TCNQ complex **7** (Figure 4) was obtained as two crystalline modifications. **7** (2:1) as prisms were synthesized from CH_3_CN-CH_2_Cl_2_ at −180 °C, whereas **7** (4:1) as plates were synthesized from MeOH-CH_2_Cl_2_ at −180 °C. In the crystal structure of **7** (2:1), a TCNQ and a cyclophane are superimposed. In addition, the benzene ring and the TCNQ of the cyclophane partially overlap, a cyclophane and a TCNQ are stacked with the D-A distance being 3.26 Å. The average transannular distance between these two benzene rings is 3.12 Å (Figure 5). Because of the disorder of the molecule, cyclophanes are found to have two conformations: *C_s_* and *C_3h_*. Each cyclophane interacts with TCNQ on only one surface of the molecule. The final consequence is that two cyclophanes are coordinated to one TCNQ. In the crystal structure of **7** (4:1), a similar D-A overlap was found in a TCNQ and cyclophane, and 3.39 Å is their D-A distance. By the force of CH-π, the other naphthene interacts (2.90 Å) with an alkene proton of TCNQ to form a 4:1 unit (Figure 5). In the crystal structure, the cyclophane moiety of [3_3_](1,3,5)CP-TCNQ-F4 (1:1) complex **8** (Figure 4) is *C_s_*. The D-A overlapping pattern is semblable to that of **7**, and 3.32 Å is the transannular D-A distance. Sectional overlapping receptors are regularly stacked with a distance of 2.93 Å in infinite columns (Figure 5). By contrast, it is possible that complex **8** has an infinite column of partially overlapping receptors with short A-A distances, and that receptors arranged parallel to each other in the plane of the unit cell are in close contact. Resulting in greater charge separation, making it more conductive.

**Figure 6:**
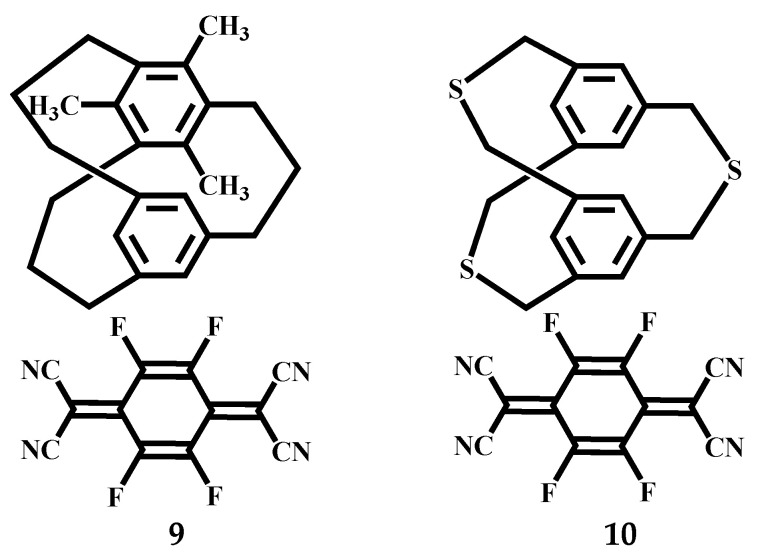
The structures of Me_3_[3_3_]CP-, S_3_[3_3_](1,3,5)CP- TCNQ-F4 complexes **9** and **10**.

**Figure 7:**
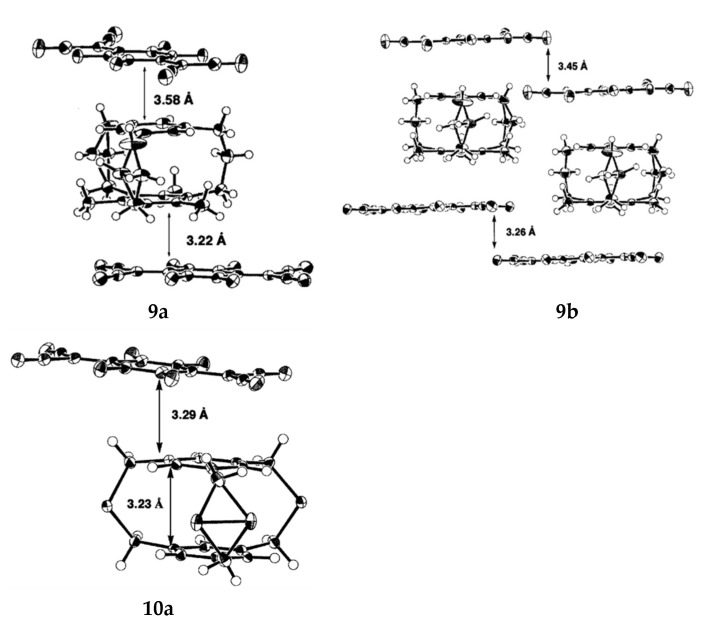
ORTEP drawings of complexes **9** and **10** at −170 °C (the probability of thermal ellipsoid is 50%).

Mikio et al. studied the solid-state structure of the Charge-Transfer Complexes of 5,7,9-Trimethyl and 2,11,20-Trithia [3_3_](1,3,5)CPs [55]. Me_3_[3_3_] CP exists in the *C_3_* conformation in both solid and liquid states, with three bridges pointing in either clockwise or counterclockwise directions. Under the reaction at −170 °C, two types of D-A overlap were found in crystals of Me_3_[3_3_](1,3,5) CP-TCNQ-F4 (1:1) complex **9** (Figure 6). One is attributed to efficient HOMO-LUMO orbital interaction, with a partial D-A overlap of the methyl-substituted benzene ring and TCNQ-F4 in the [3_3_](1,3,5)CP-TCNQ-F4 complex. In contrast, the methyl-unsubstituted benzene ring is almost completely overlapped with TCNQ-F4. The cross-ring D-A distance of the former (3.22 Å) is much shorter than the cross-ring D-A distance of the latter (3.58 Å), which illustrates the view that the methyl-substituted benzene ring interacts more strongly with the acceptor than the methyl-unsubstituted benzene ring (Figure 7). Recipients and donors are stacked alternately, and the transannular distance between recipients is 3.26 Å on the methyl-substituted side and 3.45 Å on the methyl-unsubstituted side (Figure 7). Under the reaction at −170 °C, *C_s_* structure was observed in the cyclophane portion of S_3_[3_3_](1,3,5) CP-TCNQ-F4 (1:1) complex **10** (Figure 6). The cross-ring D-A distance is 3.29 Å, and the average cross-ring distance between two benzene rings is 3.23 Å (Figure 7). Among them, partially overlapping receptors form an infinite column, and the distance between receptors is 3.10 Å, which is larger than that of complex **8** (2.93 Å), which is attributed to the weaker donation ability of S_3_[3_3_](1,3,5)CP than [3_3_](1,3,5)CP.

**Figure 8:**
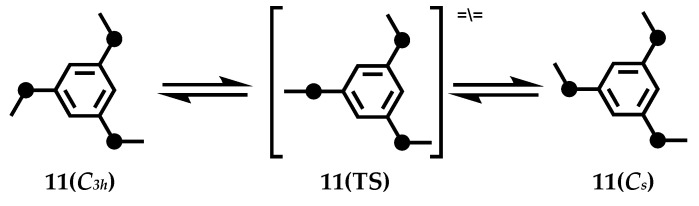
Bridge flipping process and stable conformers of [3_3_](1,3,5)CP **11**.

**Figure 9:**
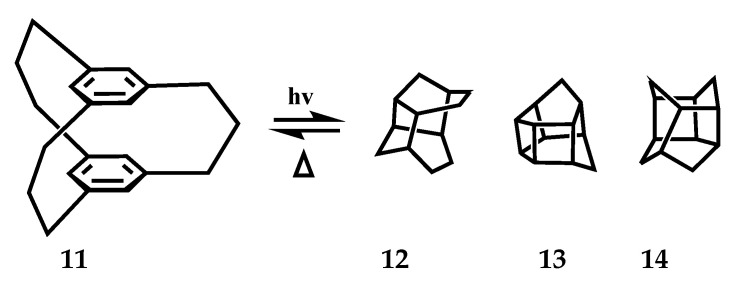
Compound **11** generates a photochemical reaction formula of the new cage compounds **12**, **13,** and **14** to show only the cage backbone for clarity.

[3_3_](1, 3, 5)CP **11** observed two conformations with symmetry of *C_3h_* and *C_s_* in the 1H NMR spectrum of 2,2,11,11,20,20-hexa-deuterated in CD_2_Cl_2_. **11** (*C_s_*) is more stable than **11** (*C_3h_*) by 0.4 kcal/mol. When the bridge is flipped, its energy barrier is 12.4 kcal/mol (Figure 8). The transition state **11** (TS) connecting the *C_s_* and *C_3h_* conformations was estimated by density functional calculation (B3LYP) to have only one plane bridge with a dihedral Angle calculated as 180.0°. One of the three bridges can transform conformation independently of the other two bridges. In the solid state, the benzene rings of **11** are completely stacked with a cross-ring distance of 3.08–3.24 Å, resulting in a *C_s_* conformation. Rika et al. studied the photochemical reactions of **11** under a sterilized lamp (254 nm) [56]. In the presence of 2 mol/L aqueous HCl solution, after photolysis in MeOH or CH_2_Cl_2_, the cyclophanes [3_3_] obtain new caged compounds and produce new skeletons, such as pentacyclo[6.4.0.0.^3,6^0.^4,11^0^5,10^] dodecane skeleton **12**, hexacyclo[6.4.0.0.^2,6^0.^4,1^10.^5,1^0^9,12^] dodecane **13** and tetracyclo [6.3.1.0.^2,7^0^4,11^]dodeca-5,9-diene **14** (Figure 9).

**Figure 10:**
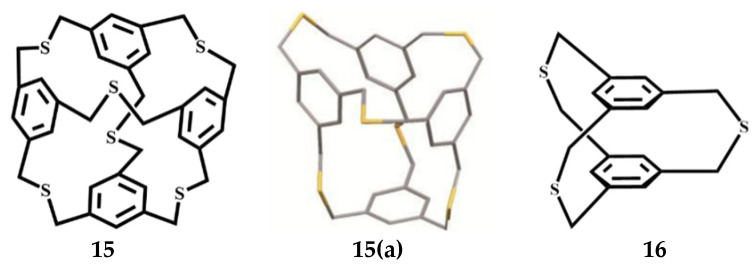
The conformation of C_36_H_36_S_6_ **15** and 2, 11, 20-trithia[3_3_](1, 3, 5)cyclophane **16**.

To investigate the structure and cation–π interactions of the metal ion complex, Hiroyuki et al. synthesized a cage-like compound C_36_H_36_S_6_ [57]. In solution, the 1H and 13C NMR spectra showed two or three sharp singlets, respectively, which suggests its symmetrical cage structure. Thus, the inclusive space is satisfyingly retained. However, by crystallographic analysis, the structure of C_36_H_36_S_6_ **15** (Figure 10) in the solid state was not symmetrical but in a collapsed shape (**15a**). X-ray crystallographic analysis revealed that the compound had a collapsed structure 2,11,20-trithia[3_3_](1,3,5)CP **16** (Figure 10). 

**Figure 11:**
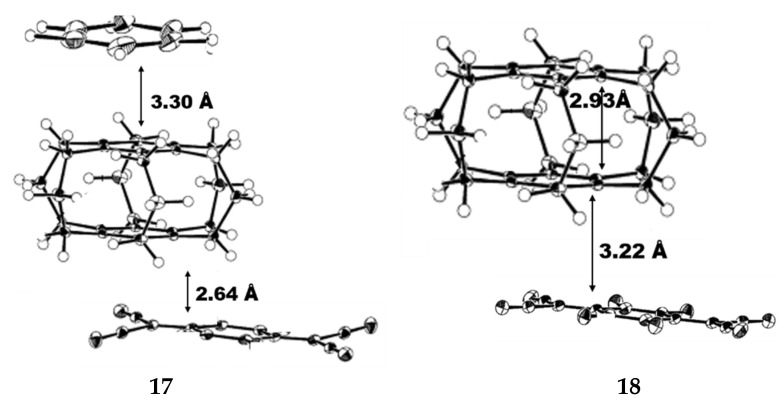
ORTEP drawings of complexes **17** and **18** at −190 °C (the probability of thermal ellipsoid is 50%).

Mikio et al. studied the crystal structure of [3_6_](1,2,3,4,5,6)CP with a series of complexes synthesized by TCNQ [58]. In the crystal structure of the [3_6_](1,2,3,4,5,6)CP-TCNQ-benzene (1:1:1) complex **17** (Figure 11) at −190 °C, a TCNQ (acceptor) and a cyclophane(donor) are stacked together with a D-A distance of 3.30 Å at −190 °C. The benzene ring of cyclophane partially overlaps with the six-membered ring of TCNQ. The short distance (2.64 Å) between an olefinic proton of the TCNQ and a benzene ring suggests the presence of a CH–π interaction. The cyclophane moiety is observed as a *D_6h_* conformer, because of the disorder of molecules with a *C_6h_* symmetry. The two neighboring cyclophane molecules face in an orthogonal position in the crystals of free [3_6_](1,2,3,4,5,6)CP, and this arrangement is generally observed in the crystal structures of [3_n_]CP. The cyclophane moiety of the [3_6_](1,2,3,4,5,6)CP-TCNQ-F4 (1:1) complex **18** takes the *C_6h_* structure in the crystals, the transannular D-A distance is 3.22 Å, the average transannular distance between two benzene rings is 2.93 Å (Figure 11). The complex shows alternating do-nor-acceptor superposition resulting in the partial donor-acceptor overlap. This structural feature can be explained by the orbital interaction between the acceptor LUMO and the donor HOMO.

**Figure 12:**
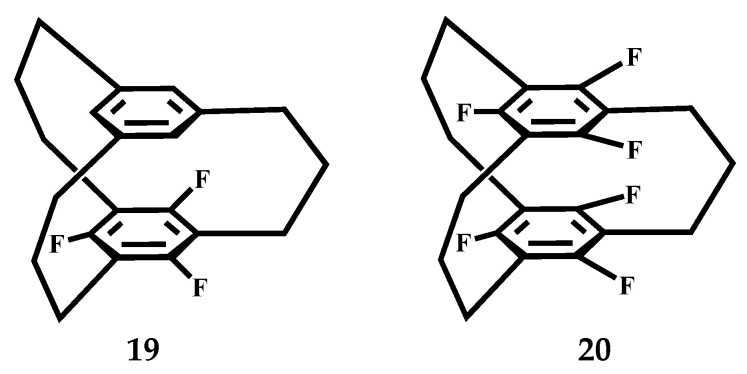
The conformation of Trifluoro- and hexafluoro [3_3_](1,3,5)CP **19** and **20**.

Koga et al. synthesized trifluoro- and hexafluoro[3_3_](1,3,5)CP **19**, **20** (Figure 12) and studied their crystal structure [59]. Their structures exist in the Cs conformation in the crystalline state, and the molecules can be stacked face-to-face with the fluorinated benzene ring to form a column. In all nonfluorinated [3_n_]CPs (n = 3–6), the two neighboring molecules are perpendicular, which is a structural feature of fluorinated derivatives. Therefore, the H–F class hydrogen bond interaction is more significant in **20** than in **19**. The longest π-π* absorption band shows a blue shift as the number of fluorine atoms increases. 

#### 2.1.2. Non-Carbon Chain Triple Cage

**Figure 13:**
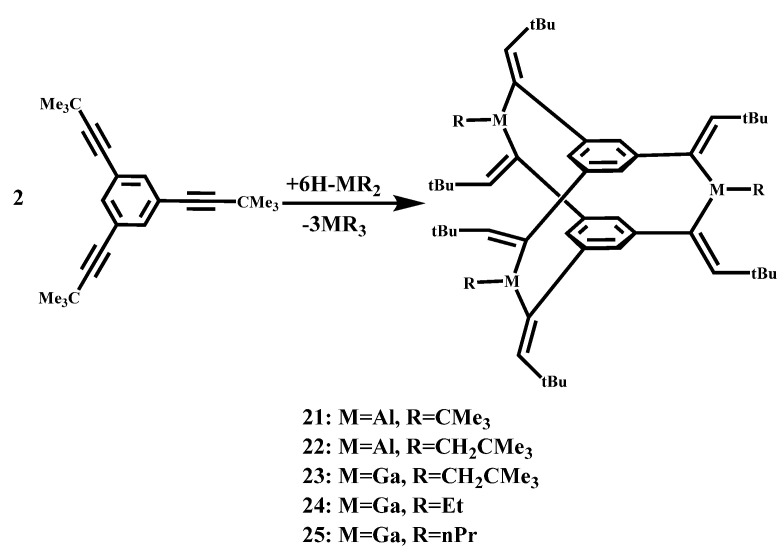
Synthesis of Aluminum-Bridged and Gallium-Bridged [3,3,3] cyclophanes derivatives.

**Figure 14:**
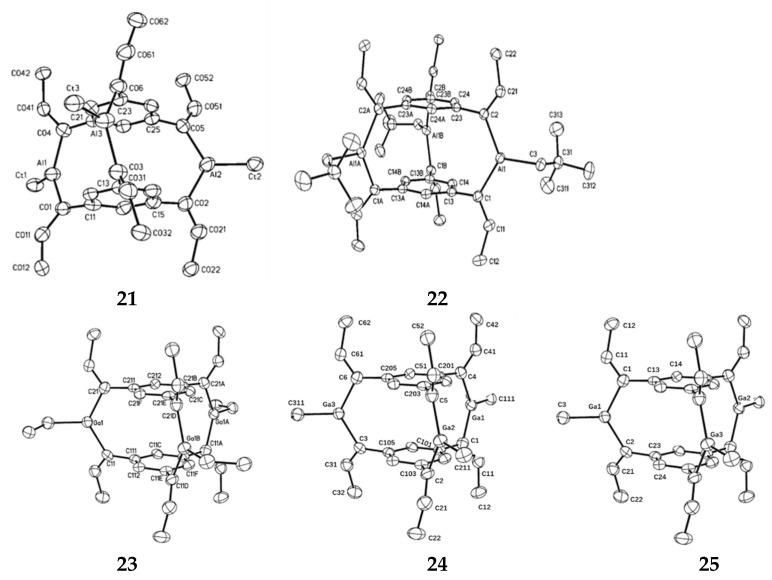
ORTEP drawings of complexes **21**, **22**, **23**, **24**, and **25** (the probability of thermal ellipsoid is 50%).

Werner et al. studied the crystal structure of Aluminum-bridged [3,3,3] cyclophanes, firstly the reaction of 1,3,5-tris(3,3-dimethyl-1-butenyl)benzene C_6_H_3_(C≡C–CMe_3_)_3_ with the dialkyl aluminum hydrides HAl(CMe_3_)_2_ and HAl(CH_2_CMe_3_)_2_ gave the addition of one Al–H bond to each C–C triple bond (hydroalumination) [60]. The reaction equation is shown in Figure 13. Spontaneous condensation by the release of the corresponding trialkyl aluminum derivatives afforded [3,3,3] cyclophane derivatives **21** and **22** (Figure 14) in which three tricoordinate Al atoms are in the bridging positions between two functionalized benzene rings. The resonances of the vinylic hydrogen atoms and the protons attached to the aromatic ring are close together and resonate at δ = 6.00 and 6.32 ppm for **21** and δ = 6.24 and 6.22 ppm for **22**. The carbon atoms of the C=C bonds show chemical shifts of δ = 152 and 156 ppm on average; the resonances at higher fields belong to the carbon atoms attached to the phenyl rings and the aluminum atoms. Due to the addition of Al–H bond, the initial C≡C bond produces an alkenyl group containing C=C bond, which decreases. The aluminum atom specifically attacks the triple-bonded carbon atom located at the alpha position of the aromatic ring. Hydrogen and aluminum are in cis on the double bond. Trialkylaluminum forms three C-Al-C Bridges between the two aromatic rings, which contain coordinated unsaturated aluminum atoms. The Al-C distance of the terminal alkyl group is very similar to the Al-C distance of the inner cage. As a result, there was no sign of spatial stress in the cage.

Werner et al. also report that the reaction of 1,3,5-tris(3,3-dimethyl-1-butynyl)benzene, C_6_H_3_(C≡C−CMe_3_)_3_ with di(neopentyl)gallium hydride, HGa(CH_2_CMe_3_)_2_, where each C≡C triple bond add a Ga−H bond (hydration) [61]. A [3,3,3] cyclophane derivative **23** (Figure 14) is generated by spontaneous condensation of tri (neopentyl) gallium, in which three trip coordination Ga atoms are located in bridging positions. The reaction equation is shown in Figure 13, with three carbon atoms bound to gallium atoms on a triangular planar coordination sphere. All configurations on the C=C double bond correspond to the addition of the GA–H bond cis to the alkyne group. Owing to the crystallographic symmetry, the two benzene rings are coplanar in the ideal state. Instead of the desired overlapping arrangement, they are slightly rotated 4.2° from each other. The R-carbon atom of the C=C double bond deviates only 0.05–0.08 Å from the average plane of the corresponding phenyl group. This small bending of the organic skeleton of a molecule is due to special bonding within the ring-sealed cage. By the reactions of tris(tert-butyl ethyl) benzene with dialkyl gallium hydride Werner et al. also obtained two cyclophane-type molecules **24** and **25** (Figure 14) with three gallium atoms at the bridging position between the two benzene rings [62], the reaction equation is shown in Figure 13. The change in the number of bridge groups does not greatly affect the structural parameters. The C=C double bond length is in good agreement with the standard value, which is about 1.34 Å on average. The C-Ga-C angle in the cage (123°) is slightly increased relative to the ideal value of the *sp*^2^ atom, but this may be due to some strain reaction in the molecule. A larger angle of 131° to 134° was observed for C=C-CMe_3_. They may be caused by interactions between the aromatic system and tert-butyl groups, or they may be caused by spatial interactions between tert-butyl groups arranged on a benzene ring.

#### 2.1.3. Triimidazole Bridge Cage

**Figure 15:**
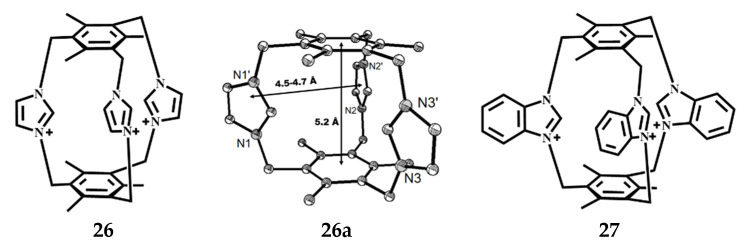
The structures of cyclophanes **26** and **27** and ORTEP drawings of complex **26** (the probability of thermal ellipsoid is 50%).

Yi et al. reported the convenient and highly effective synthesis method of novel water-soluble tris-bridged imidazoles containing imidazolium or benzimidazolium groups **26**, **27** (Figure 15) [63]. Analysis by X-ray showed that compound **26** has *C_s_* symmetry. The symmetry mirror is the plane of three 2-C of imidazolium rings. The parallel capping benzene rings are about 5.2 Å apart. The distances of the three 2-C of the imidazolium are 4.5–4.7 Å (Figure 15). The rigidity, size, and shape of cyclophanes **26** and **27** make them suitable for the formation of supramolecular systems containing complex small anions alone.

**Figure 16:**
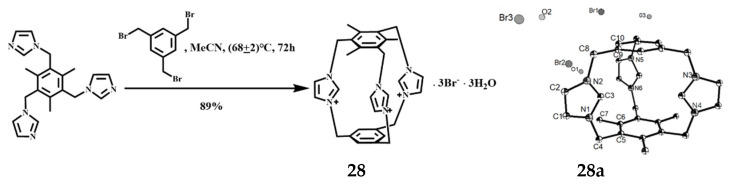
Synthesis and ORTEP drawing of a speleand imidazolium cyclophane **28** (the probability of thermal ellipsoid is 50%).

Yuan et al. obtained a speleand imidazolium cyclophane [C_30_H_33_N_6_]^3+^·3Br^−^·3H_2_O] **28** by direct quaterization of 1,3,5-trimethyl-2,4,6-tri (imidazolylmethyl) benzene with m-tri(bromomethyl)benzene and its yield is 89% [50], the reaction is shown in Figure 16. The repeating crystal structural unit consists of one large bicyclic triimidazole cyclopean, three bromine anions, and three water molecules. The three imidazole rings and the two benzene rings are surrounded by a cavity, the three imidazole rings are asymmetrically arranged, and there is no symmetry factor in the molecule itself, but the imidazole ring is homogenized, and the positive charge is dispersed on the imidazole ring. Outside the cavity, the nest formed between the imidazole rings can also be used to selectively bind the molecule to anionic or neutral molecules by electrostatic action and cationic 2π interaction. The molecules have a strong rigidity, due to the presence of methylene, the angle between the imidazole rings has a certain degree of tunableness, so that the molecules have a certain large double-ring effect, and can be selectively combined with inorganic, organic anions, or amphoteric and neutral molecules to form a supramolecular system. Preliminary studies have shown that the compound has a good selection and recognition effect on some common anions and polyphenols.

**Figure 17:**
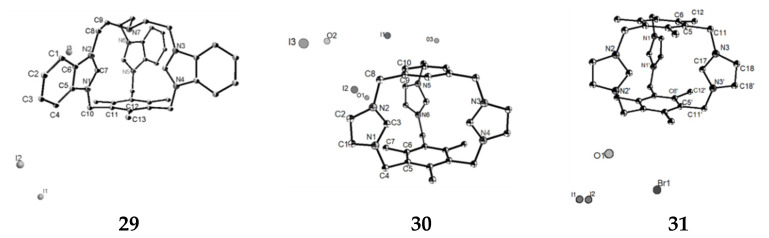
ORTEP drawings of cages **29**, **30** and **31** (the probability of thermal ellipsoid is 50%).

We synthesized three 3D imidazole or benzimidazole cages **29** (L1·I_3_), **30** (L2·I_3_), and **31** (L3·Br_2_·I) as trivalent cation templates and studied their cage structures (Figure 17). Free asymmetric cage **29** with I^-^ only through the weak interaction. Two independent conformations can be found in the crystal structure. The dihedral angles between the benzimidazole rings are 24.53°, 70.44°, and 85.04° in type A, respectively. In type B, the three dihedral angles are 17.03°, 77.25°, and 88.73°, respectively. This indicates that the conformations of the two cages of **29** are different, but they can be changed to some extent. In the structure of asymmetric cage **30**, the two benzene rings are almost parallel, but **30** has a symmetric mirror image, and the plane of symmetry is the plane of the three 2C atoms of the imidazole group. **30** belongs to a class of cylindrical macrocyclic salts containing the imidazole family; the shape of the compound makes it suitable for the formation of exclusive or included complexes with small anions. The structure of **31** is different from that of **30**, which is symmetric, but the size of the cage cavity is similar to that of **30**. 

**Figure 18:**
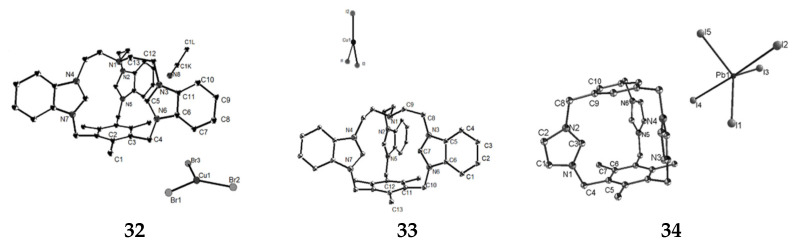

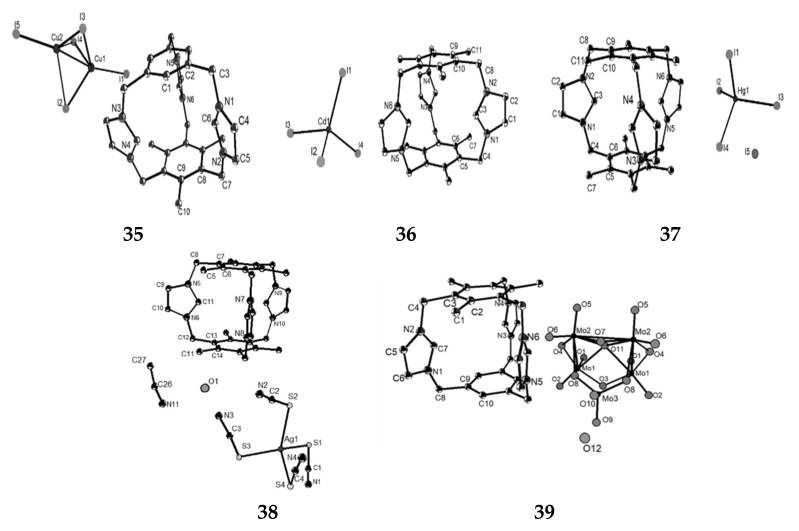
ORTEP drawings of eight different organic-inorganic supramolecular hybrids (the probability of thermal ellipsoid is 50%).

We used **29–31** to assembly with metal (pseudo) halides or molybdates and obtained eight different organic-inorganic supramolecular hybrids: {(L1)2·[CuBr_3_]_3_·2(CH_3_CN)}**32**, {L1·[CuI_3_](H_2_O)}**33**, {L2·[PbI_5_]}**34**, {(L2)_2_·[Cu_2_I_5_]_2_}**35**, {(L3)_2_·[CdI_4_]_3_}**36**, {L3·[HgI_4_]·I}**37** {L3·Ag(SCN)4·(CH_3_CN)·2H_2_O}**38** and {L2·[HMo_5_O_17_](H_2_O)_3.25_}**39** [64,65], ORTEP drawings are shown in Figure 18. Their structures were determined by single-crystal X-ray diffraction analyses. The structure of compound **32** exhibits conformational contraction and the distance between the benzene ring and the nitrogen atom on ethylamine is 4.909 Å. The dihedral angles of the two benzimidazole rings are 64.93°, 19.70°, and 45.49°, respectively, due to the electrostatic force generated by the cage and the metal halide. **33** and **32** are identical in that the structure consists of **29** and Cu(I) but differ in the anion composition. Due to the hydrogen bond interaction and the electrostatic force between **29** and the metal halide, the vertical height of the cation cage of **33** is shorter than that of the free state **29**. In the structure of compound **34**, the three imidazole rings of the cationic cage are not symmetric, which is attributed to the induction interaction between the cationic cage and [PbI_5_]^3−^, which changes the structure of **34**, resulting in a shorter vertical height of **34** than the free **30**. In the structure of compound **35**, due to the interaction between the cationic cage and the strong Cu-Cu anion in the binuclear cluster species, there are two conformations in **35**, in which the two cationic and anion parts are not the same, resulting in the benzene ring distance of 5.1905 Å and 5.1415 Å, respectively. These results indicated that the two cages exhibited different breathing behaviors of contraction and expansion. In this structure of compound **36**, the two triimidazole cage cations in the structural unit are different. Different metal halide anions are present in **37** compared with compound **36**. However, the anions in both compounds showed similar compressive effects on the cationic cage. Compound **38** contains strong C–H···N hydrogen bonds, three imidazole rings asymmetrically arranged. In this structure of compound **39**, a stacking pattern formed in the c direction due to the action of weak electrostatic forces. In addition, by studying their fluorescence characteristics in the solid state, it is shown that they can be used as fluorescence sensors for detecting Fe^3+^ ions and have high recyclability and sensitivity.

Our previous research has shown that cationic conformations are significant in anion assembly and final architecture [64]. Tris (imidazole) ring cage **29–31** has the characteristics of insufficient internal space and strong rigidity and is looked forward to inhibiting the template effect to induce or contain anionic guests. Mononuclear or polynuclear anionic structures (compounds **32–38**) are generally composed of cationic cages assembled with electron-deficient metal halides. However, when assembled with electron-rich polyoxometalates (POMs), a rare [Mo_5_O_17_]^4−^ anion isomer is produced (compound **39**). Thus, the retention of a 1,3,5-trialkylbenzene cation cap with a special structure may have a typical template effect on electron-rich polyoxometallates. Negatively charged species can also direct the formation of specific molecules and compositions [66]. It is interesting to observe the triimidazole cage structure from an anionic perspective. Although triimidazole ring cages have a lot to do with anions or anionic clusters, they can only regulate a limited amount of space to accommodate different types of anions. Therefore, the vertical height of the cage can be finely adjusted for the conformation. After complexation with polyoxometalates, the cage-like structure in compound **39** expands (~0.07 Å); For metal halides, this complexation leads to shrinkage of the cage (~0.01–0.1 Å at **32–38**) or expansion (~0.04 Å at **39**). This mechanism can be described as the “breathing process”. These results show that these rigid cages have certain mobility that is comparable to the conformational changes of the vehicle-pile complexes below (~0.16 Å) [67]. 

**Figure 19:**
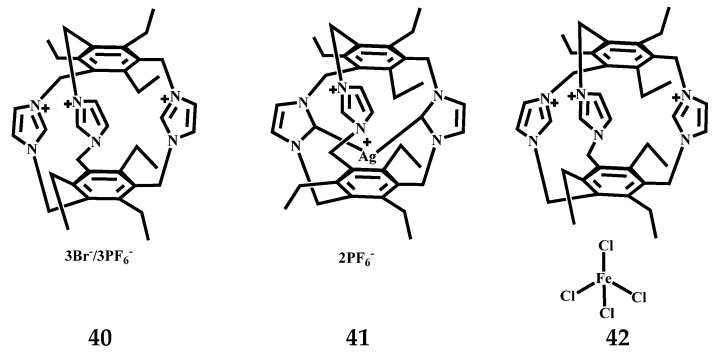
The structures of tris(imidazolium) cyclophane **40** and its two organic-inorganic supramolecular hybrids.

Willans et al. reported sterically rigid tris(imidazolium) cyclophane **40** (L4) (Figure 19) reacts with Ag_2_O to give an Ag(I) mononuclear carbene complex [Ag(L4-2H)](PF_6_)_2_ **41** (Figure 19) characterized by X-ray crystallography [68], which revealed a solid-state structure entirely consistent with the solution NMR spectroscopic data. The C–Ag–C angle of the complex is 175.9°, which is linear. The structure exhibits almost perfect specular symmetry, but the slight rotation of the remaining imidazole perpendicular to the C–Ag–C axis breaks the rotation. This change may be attributed to the same resonance of H in acetonitrile, which can be well explained by the detection of NMR spectroscopy, which also shows that the complex is rigid, and the close contact between the Ag(I) central imidazole and CH groups is repulsive, thus increasing the activation barrier of exchange. The centroid separation of the aromatic ring increases from 5.15 Å in the structure of **31** to 5.31 Å due to the axial property of C–Ag–C, which leads to the expansion of the complex. The resulting rigid complex is stable to water and air and exhibits well-defined conformational properties. The reaction of 1,3,5-trimethylimidazole-2,4,6-triethylbenzene with FeCl_3_·6H_2_O in hot water solution resulted in the isolation of L4 as the mixed bromide–tetrachloroferrate (III) salt, L4^3+^Br^2^ · 2[FeCl_4_]_2_ **42** (Figure 19). Salt **42** adopts a threefold symmetric cubic packing arrangement in the solid state. The molecular structure of the L4^3+^ cation is similar to that of the L3 bromide, although the aromatic intercentroid separation time is shorter (5.08 Å). L4^3+^ and two independent [FeCl_4_] anions are both located on the crystallographic triplet axis, and a chloride ligand penetrates the cage produced by the ethyl substituent to form a very short anion–π interaction with a Cl_3_Fe-Cl···π distance of 3.424 Å, which corresponds to the sum of van der Waals radii. As a result, the triple axis by L4^3+^···[FeCl_4_]_2_···[FeCl_4_]_2_···Br_2_···L4^3+^ polarity chain as the packing, etc. The addition of Br^-^ allows the structure to avoid the mismatch between the cyclomatic hydrocarbon and the triangular face of the [FeCl_4_] anion. That is, bromine incorporation is necessary because the cation has an amorphous mirror symmetry perpendicular to the triple axis of the molecule and the crystal, whereas the tetrahedral anion does not. This co-existence of highly symmetric matching and hindrance provides a possible strategy for the design of polar molecular crystals.

### 2.2. Crystalline Four-Fold Bridged Cyclophanes

**Figure 20:**
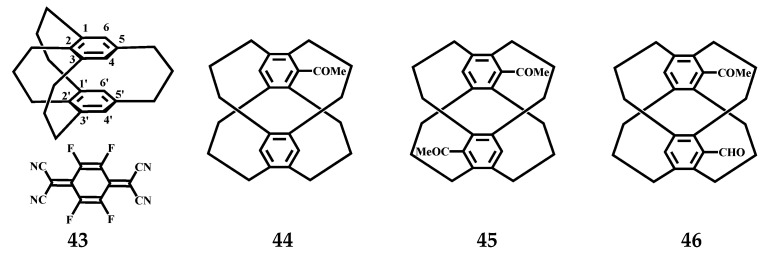
The structures of [3_4_]CP-TCNQ-F4 complex **43** and the monoacetyl and diacetyl compounds **44**, **45** and **46**.

Mikio et al. estimated by Molecular Orbital calculations that the most stable conformation of [3_4_](1,2,3,5)CP crystals at −150 °C is the *C_s_* structure. The transannular distance between the two benzene rings of cyclophane is 3.198–2.978 Å [37]. The distance between C4-C4′ without bridge (3.198 Å) is much longer than that between C1-C1′ with bridge (2.978 Å) (Figure 20). These data illustrate certain variations of the benzene rings of [3_4_](1,2,3,5)CP. In addition, [3_4_](1,2,3,5)CP-TCNQ-F4 (1:1) complex **43** (Figure 20) shows the *C_s_* structure in the crystal. The crystal structure of pseudogemstone-acetylformyl [3_4_](1,2,3,5)CP, a significant synthetic intermediate in aldol reaction, was also determined. The benzene ring was perpendicular to the attached acetyl group, the formyl group was located in the plane of the attached benzene ring, and the carbonyl oxygen was oriented to the inner side of the molecule. In bright comparison to the molecular structures of the monoacetyl compound 5-acetyl[3_4_](1,2,4,5)CP **44** and the diacetyl compound 5,18-diacetyl [3_4_](1,2,4,5)CP **45**, the two benzene rings are completely stacked in pseudogem-acetylformyl [3_4_](1,2,4,5)CP **46** (Figure 20).

**Figure 21:**
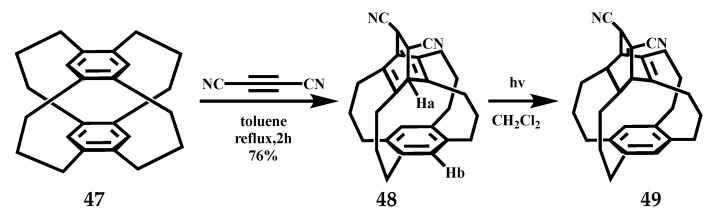
The cycloaddition and photoirradiation of [3_4_](1,2,4,5)CP **47**.

Wakana et al. reported the modified synthetic route to [3_4_](1,2,4,5)CP **47** provide gram quantities of this compound in fewer steps than the conventional routes [69]. The cycloaddition of [3_4_](1,2,4,5)CP with dicyanoacetylene gave barrelenophane **48**, which was transformed into semibullvalenophane **49** on photoirradiation, the reaction formula is shown in Figure 21. The X-ray structural analyses of **47**, **48,** and **49** demonstrate their unique structures. The geometry of the benzene ring of **47** is bent from planar to a boat-shaped form, the molecule assumes the most stable *D_2h_* structure. In the structural diagram of barrelenophane **48**, four trimethylene bridges assume the boat–boat conformation, and a similar boat-shaped deformation of the benzene ring was also found. Tetra-bridge cyclohexanol **48** has a low strain and rigid structure. Compound **49** is the structure of the semibullvalenophane, the benzene ring of **49** is slightly deformed into the boat form and the distortion angle α is 9.89°. The cyclopropane ring of **49** is significantly distorted from a regular triangle.

**Figure 22:**
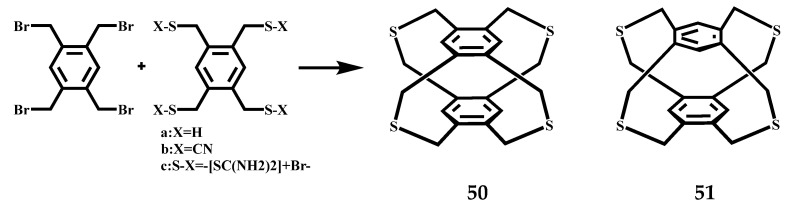
Synthesis of tetrathiacyclophanes **50** and **51**.

Toshiaki et al. have examined the coupling conditions of tetrabromide with three kinds of tetrasubstituted durene derivatives, to optimize the synthesis method of tetrathiacyclophanes **50** and **51** [70], the reaction formula is shown in Figure 22. Compound **50** shows a *D_2h_* symmetric isomer and compound **51** shows a *C_2v_* symmetric isomer in the crystal structures. Their structural diagram shows four -CH_2_-S-CH_2_- bridge conformations, in which all the sulfur atoms point to the unsubstituted aromatic hydrogen. The two rings may slide in parallel to reduce repulsive forces.

**Figure 23:**
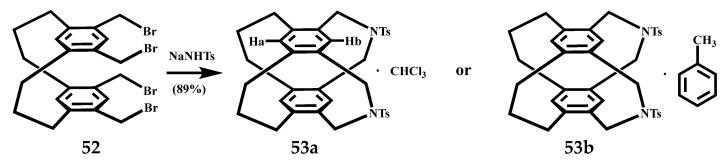
Synthesis of a new quadruple bridged cyclophane **53**.

Stefan et al. cyclized the quadruple functionalized cyclophane **52** with the toxamide monosodium salt to obtain a new quadruple bridged cyclophane **53** [71], the reaction formula is shown in Figure 23. The crystal structures of **52** and **53** were analyzed by X-ray. Although the structure of **52** contains four bromomethyl groups, it still shows a typical cyclophane conformation. Two of the bromomethyl groups point into the interior of the cyclophane, which is caused by crystal packing. In addition, the zig-zag arrangement of cyclophanes in the crystal structure may be due to intermolecular bromine–bromine interactions. Each **53a** structure contains a molecule of trichloromethane, and each host molecule forms a hydrogen bond with a sulfonyl group. The phenyl propyl group and the second phenyl propyl group of the adjacent molecule will form an H bond and have a weak interaction. The conformation of the first nitrogenous bridge forming the hydrogen bond is ship–ship, while the second nitrogenous bridge is chair-shaped and positively intersected with the cyclophane skeleton. Crystal **53b** was obtained from the toluene solution, and each host molecule contained two solvent molecules. The first toluene molecule acted as a tweezer and formed X-donor through CH–X interaction. The other toluene molecule plays the role of inclusion and does not bind to other molecules. It exists between the two crystal layers and shows weak interaction with the cyclophane molecules in each layer. X-ray analysis showed that the crystallization of **53** in two different solvents resulted in a conformational change in the guest, with crystal **53a** containing trichloromethane having a boat conformation with a *C_s_* symmetry, whereas crystal **53b** containing toluene had a chair conformation with a *C_2v_* symmetry. Through a structural comparison of the two, it was found that different sizes and types of molecular bands and molecular tubes could be obtained by changing the building blocks in the synthesis strategy.

**Figure 24:**
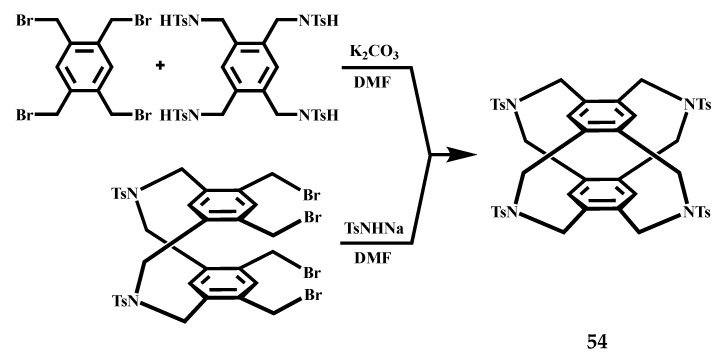
Synthesis of the fourfold-bridged cyclophane **54**.

Josten et al. prepared the fourfold-bridged cyclophane **54** by intramolecular and intermolecular cyclization reactions [72], the reaction formula is shown in Figure 24. The X-ray structure analyses of **54** show S-shaped conformations of the molecules. By analyzing its single crystal structure, studied the crystallization of Aza[3.3]-supercyclone in a synchronous conformation and had a characteristic sequence of boat/armchair conformations in bridges.

### 2.3. Crystalline Five-Fold Bridged Cyclophanes

**Figure 25:**
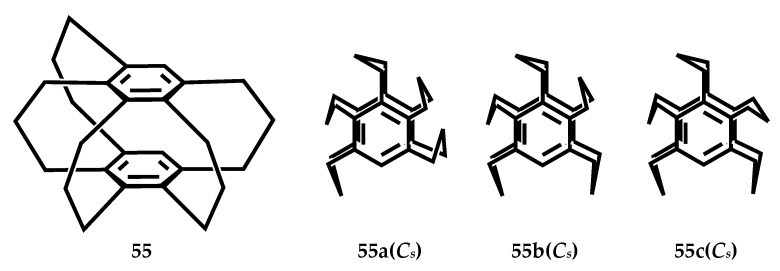
The stable conformers of **55a**, **55b** and **55c** of the [3_5_]CP **55**.

**Figure 26:**
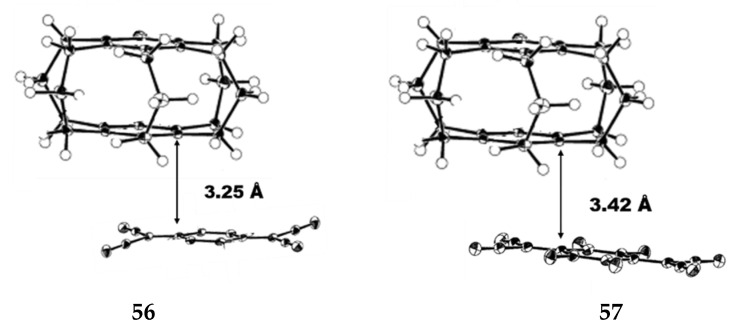
ORTEP drawings of complex **56** and **57** at −180 °C (the probability of thermal ellipsoid is 50%).

**Figure 27:**
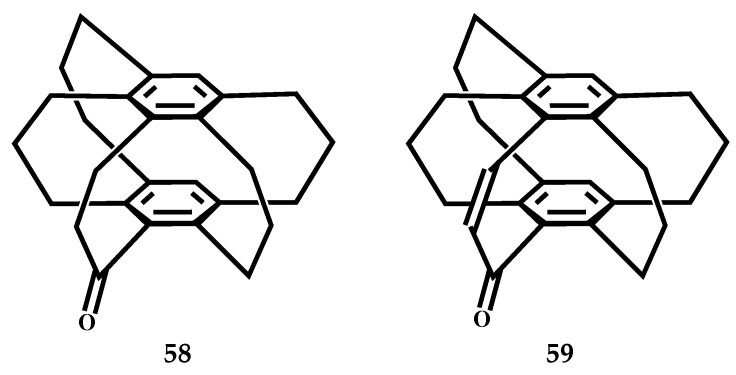
The structures of [3_5_](1,2,3,4,5)CP-1-one **58** and **59**.

Mikio et al. studied the structure of [3_5_](1,2,3,4,5)CP **55**, as shown in Figure 25, **55b** is the most stable conformation of the three [3_5_]CPs [37]. At −180 °C, in the crystal structure of the [3_5_]CP-TCNQ (1:1) complex **56**, its D-A distance is 3.25 Å (Figure 26), complex **57** (2:1) and [3_6_](1,2,3,4,5,6)CP-TCNQ-F4 (1:1) complex **18** similar to that of D-A overlapping structure. The distance between the acceptors in the unit cell is 3.42 Å (Figure 26). The cyclophane structure of CP-TCNQ adopts the *C_s_* conformation, and the disorder of the bridge is observed, which is related to the semblable stability of 55a and **55b** conformations. The enone bridge of **58** (Figure 27) has a planar structure, and the two adjacent trimethyl bridges are far away from the enone bridge. [3_5_](1,2,3,4,5)-CP-1-one **59** (Figure 27) crystal structure is similar to [3_5_]CP **55**. The cross-ring distance between the benzene carbon and the COCH_2_CH_2_- group was 2.93 Å, which was shorter than the other distances (3.05 and 3.22 Å).

### 2.4. Crystalline Six-Fold Bridged Cyclophanes

Mikio et al. studied the structure of [3_6_](1,2,3,4,5,6)CP. On the one hand, In the crystal structures of the [3_6_](1,2,3,4,5,6)CP-TCNQ (1:1) complex **17** (Figure 11) exhibits molecular disorder with *C_6h_* symmetry, while its cyclophane fraction is observed to be *D_6h_* structure [37]. On the other hand, the *C_6h_* structure is observed in the crystal of [3_6_](1,2,3,4,5,6)CP-TCNQ-F4 (1:1) complex **18** (Figure 11), which showed alternating donor-acceptor superposition and partial donor-acceptor overlap.

## 3. Conclusions

In summary, the conformational behavior of the four types of crystalline multiple-bridged cyclophanes: three-fold bridged cyclophanes, four-fold bridged cyclophanes, five-fold bridged cyclophanes and six-fold bridged cyclophanes is enumerated. By changing the type of aromatic compounds or regulating the type and quantity of bridging groups, multiple-bridged cyclophane cages are endowed with new self-assembly strategies and physical-chemical properties. The elongation of the bridge makes the cyclophane structure more strain-free and more flexible. It is shown that [3_n_]CPs have a stronger π-electron delivery capability than the corresponding [2_n_]CPs. The crystallization of cyclophane from different solvents may lead to guest-dependent conformational variations. By using [3_n_]CPs with strong power supply capabilities, they can be used as electron donors and electron receptors such as TCONE, TCNQ, and TCNQ-F4, so [3_n_]CPs may be donor molecular candidates with great promise for the development of new conductive electronic materials, which is of great significance for the development of electronic conductors and superconductors. 

Due to the presence of flexible alkylene bridges, the multiple-bridged molecule has strong rigidity/tunableness and can be selectively combined with inorganic, organic anion, or neutral molecule/atom to form a supramolecular host–guest system. For example, a multiple-bridged cyclophane cage consisting of benzene rings and positively charged imidazole rings can form a cavity that encapsulates some suitably sized anions. Outside the cavity, the nests formed between the imidazole rings can also be used to selectively bind the molecule to anionic or neutral molecules by electrostatic action and cationic–π interaction; whereas inserting a noble gas (Ng) atom into the cavity of the superphane molecule [2.2.2.2.2.2](1,2,3,4,5,6)cyclophane with six flexible ethylene bridges may lead to the formation of a Ng@superphane endohedral complex and the corresponding significant “swelling”, which is mainly manifested by increasing the distance between benzene rings [73]. 

The study of these crystalline multi-bridge cyclophanecages not only provides interesting examples of chemical conformations, but also provides new insights into the construction of functional solid materials. At the same time, the effects of host structure modification and different guests on the obtained supramolecular structure can be evaluated, the mechanism of host–guest framework formation can be elucidated, and the potential application of its structure–performance relationship as functional materials can be further explored.

## Data Availability

Not applicable.

## Sample Availability

Samples of the compounds are not available from the authors.
